# Supplementary material for: Experimental study on polymer friction composite with natural friction modifiers for brake pads
Source: PLoS One. 2025 Sep 8;20(9):e0328987. doi: 10.1371/journal.pone.0328987 (PMC12416750; doi:10.1371/journal.pone.0328987)
Supplement: Attachment — Submitted filename: Response to Reviewers.docx [file pone.0328987.s003.docx]

**EXPERIMENTAL STUDY ON POLYMER FRICTION COMPOSITE WITH NATURAL FRICTION MODIFIERS FOR BRAKE PADS**

**PONE- D-25-01368 [EMID:a364419a803f3350**]

The authors are grateful to the reviewers for their comments and suggestion. Below are the responses to the reviewer comments and suggestions. For your kind note, the changes in the revised manuscript have been highlighted with yellow color for convenience.

**Response to Reviewer:** Comments are in Red color and Responses are in Blue color, References in black color.

1. Paper is serving academic interests and lesser technological meaning due to tribology of natural fibres at rubbing interface.

As the material is concerned with application of brake pad where the tribology of rubbing surface holds greater significance hence paper speaks about the same.

2. Mathematical equation should be aligned in revised paper or use professional editing service for betterment of language and orientations of equations.

The suggestions have been incorporated and necessary alignment of mathematical equations have done.

3. All figures should be uniform for labelling of axis/number pattern.

Thank you for the insightful feedback. To ensure consistency in the labeling of axes and numbering patterns, we have revised all graphs to follow a standard format, with uniform axis labels, font sizes, and consistent numbering. This revision improves the clarity and professionalism of the presentation, making it easier to compare results across different figures."
